# Leg Fracture Associated with Synostosis of Interosseous Membrane During Running in A Soccer Player

**Published:** 2018-03-31

**Authors:** F. OLIVA, R. BUHARAJA, R. IUNDUSI, U. TARANTINO

**Affiliations:** 1 Department of Orthopaedics and Traumatology, University of Rome “Tor Vergata”, Rome, Italy

**Keywords:** Interosseous Membrane, Leg Fracture and Synostosis

## Abstract

**Introduction:**

Leg fractures may occur frequently in sport injuries but it is very rare to find this kind of injury associated with interosseous membrane synostosis. This case report describes a unique case of 42 B1.2 fracture of the leg associated with an interosseous membrane synostosis and literature review on Pubmed, Google scholar and Medscape.

**Case Presentation:**

A 26 year old male amateur soccer player came to our attention at the emergency room after a fall while he was running without any direct trauma following a referred ankle sprain. X-ray and CT scan of the left leg showed a comminuted displaced fracture of the lower middle third of tibial and peroneus diaphysis, and moreover, a fracture of peroneal malleolus associated with a bone bridge between the tibia and fibula. The patient was treated with a surgical osteosynthesis the day after trauma.

**Conclusion:**

We think that the interosseous membrane plays an important role in biomechanics of the leg even during running. To our knowledge, this is the first case reported which show the fractures of the tibia and fibula associated with an ipsilateral synostosis of the interosseous membrane.

**Class of evidence:**

Level V.

## INTRODUCTION

Leg fracture 42-B1.2 (AO classification)^1^, may occur frequently in sport injuries but it is very rare to find this kind of trauma associated with an interosseous membrane synostosis. The proximal and distal heterotopic ossification of the interosseous membrane between tibia and fibula (tibiofibular synostosis) is associated with ankle pain during the activity and it has been well documented 2. Proximal synostoses are correlated with leg length discrepancy and sometimes with hereditary exostoses, while distal synostoses are correlated with high ankle sprains and damage to the syndesmotic ligaments^3^. Recently an attempt of etiopathogenesis and anatomic location of leg synostosis has been published. Type I, synostosis of the distal end of the membrane as a result of a syndesmosis ankle sprain. Type II, the synostosis is present in the proximal or middle third of the membrane, the etiology was related to an old complete tibia fracture. Type III, ossification is located in the middle third of the membrane, the lesion is attributed to stress phenomenon to the interosseous membrane^4^. We used for our case the accepted anatomic O’Dwyer classification^5^. We present the case of 42-B1.2 fracture of the leg associated with an interosseous membrane synostosis in 26 year old male amateur soccer player.

## II. CASE PRESENTATION

A 26 year old male amateur soccer player came to our attention at the emergency room after a fall while he was simply running. During the physical examination the leg was swollen, hot and painful. The patients was unable to weight bearing. The range of motion of knee and the range of motion of ankle were not evaluable because of intense pain. In addition, his history and past medical history were negative for any trauma at the left leg or any tumor syndrome.

**Fig. 1:**
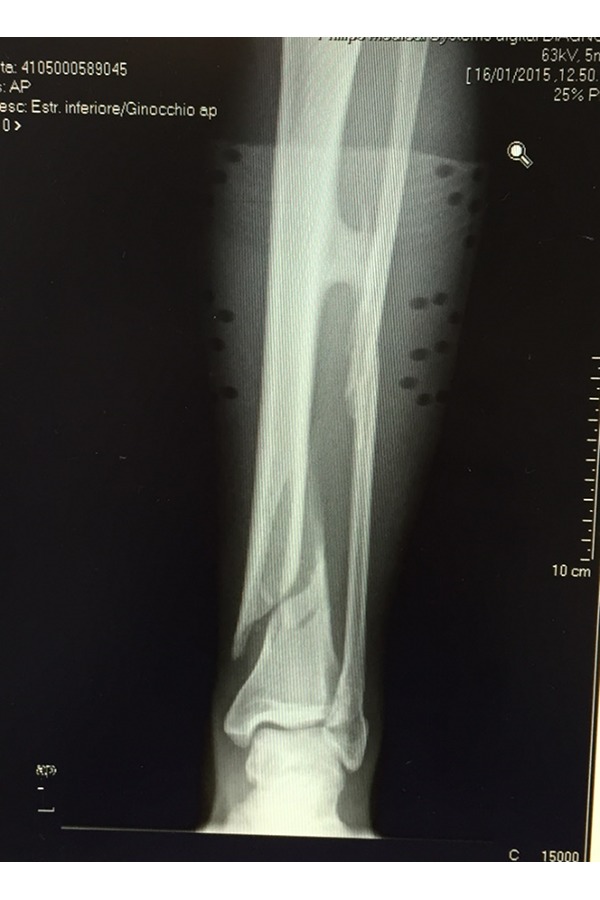
X--ray left leg shows 42 B1.2 leg fractures

**Fig. 2:**
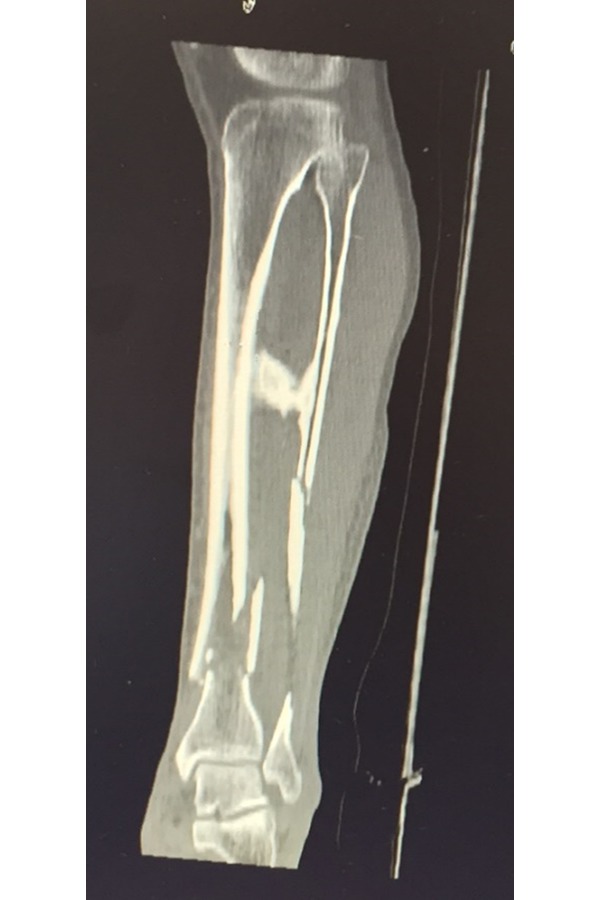
CT scan left leg confirms leg fracture and synostosis

**Fig. 3:**
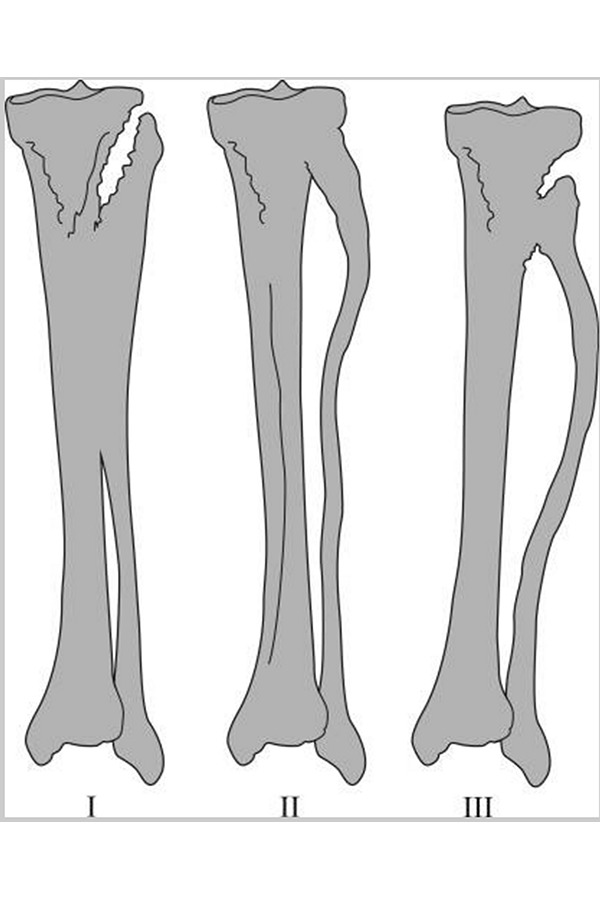
O’DWYER classification Type I Straight fibula with synostosis occurring proximally. Type II Fibula of normal length with mild bowing and widening of interosseous distance in proximal half Type III Synostosis at a more distal level than type II and marked bowing of fibula which occurs throughout its length with increased interosseous distance occurring into the distal half.

X-rays of left leg showed a comminuted displaced fracture of the lower middle third of tibial and peroneus diaphysis and a fracture of peroneal malleolus. Proximally to the diaphyseal fracture, a bone bridge between the tibia and fibula was documented (Fig. 1) Furthermore the patient was submitted to a leg CT scan which confirmed a synostosis at the middle third of the tibia that continued through the interosseous membrane up in proximity to the fibula (Fig. 2). At this point synostosis was classified as type I following O’Dwyer classification for proximal tibiofibular synostosis^5^ (Fig. 3).

**Fig. 4:**
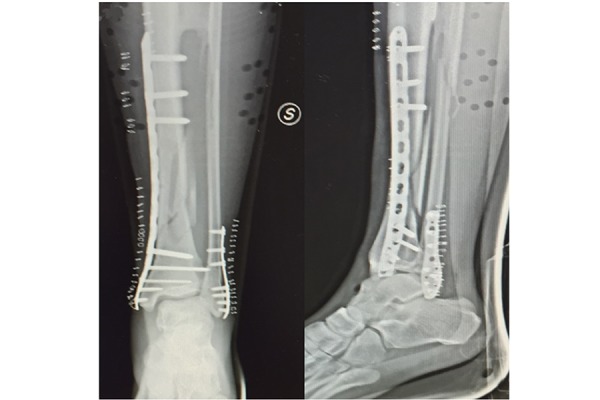
X-ray shows the final osteosynthesis

**Fig. 5:**
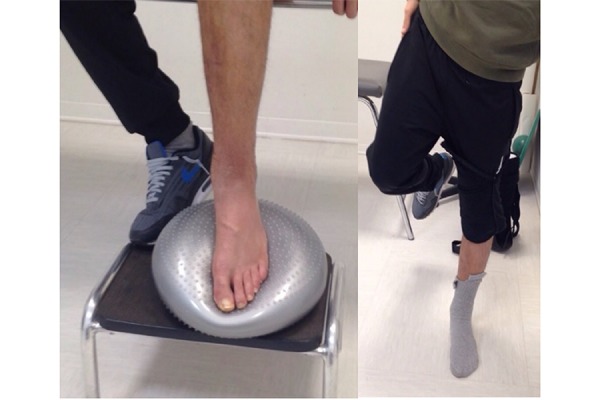
The patient during proprioceptive excercises after 6 month from the injury

**Fig. 6:**
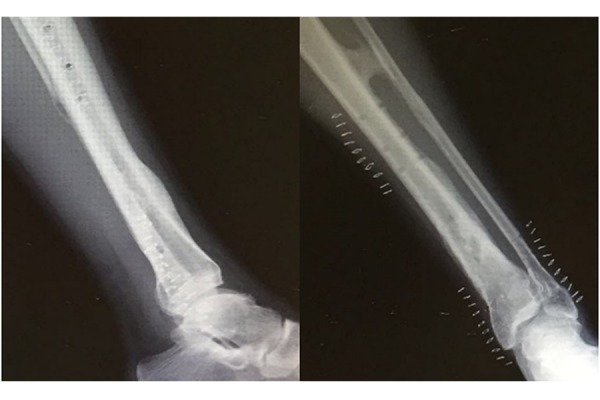
X-ray shows the removal of plates and screws after 1 year from the injury

The synostosis was defined idiopathic after excluding other possible causes of calcification of the interosseous membrane such as bone cancer and cartilage or metastatic lesion, previous trauma of the leg and osteogenesis imperfecta. The fractures were classified as 42 B1.2 following the AO classification^1^. The patient was informed about his trauma and consequently he signed the detailed consent form approved by our Institution. The day after, the patient was submitted to surgical osteosynthesis. The tibial fracture was fixed with a 3,5 mm Locking Compression Plate (LCP) (DePuy-Synthes Companies), using minimally invasive ostheosyntesis^6^. The lateral malleolus fracture was fixed with a low profile plate 1,6 mm (Response-Ortho Company), (Fig. 4). The diaphyseal peroneal fracture was not fixed. The leg was immobilized postoperation with walker cast Airselect Elite (DJO-Global) for thirty days. After 2 weeks the patient underwent to a personalized rehabilitation program by an expert physiotherapist three times per week. The rehabilitation program was composed by 3 steps (Tab. 1, Fig 5). The plates were removed after one year because the patient complained mild pain around lateral malleolus during running (Fig 6).

## III. DISCUSSION

The literature is very poor regarding cases of diaphyseal tibiofibular synostosis. We were not able to find cases in the literature (using search engine such as PubMed, Google Scholar and Medscape) describing a fracture of the leg without a major trauma in a patient with tibial-fibular synostosis. The biomechanical role of interosseous membrane is to absorb and to distribute the torsional load to the ankle joint 7. The synostosis is defined as “union between adjacent bones or parts of single bone made up of osseus material, such as ossified connecting cartilage or fibrous tissue” by Umesan. The etiology of synostosis seems to derive from a soft tissue injury with bleeding around the interosseous membrane leading to new bone formation 8.

Frick et al in their study defined the role of the synostosis concluding that the synostosis has an important role in the biomechanics of the movements between the tibia and fibula during weight-bearing^9^. The fracture of the synostosis may lead to an increased risk of developing a compartment syndrome, due to increase intramuscular pressure as suggested by studies of Hanypsiak et al.^10^ In our case, we did not observe any fracture at the level of the synostosis and there were no symptoms of a possible compartment syndrome. The synostosis is treated if symptomatic, Henry and Horst^11^ suggested conservative treatment in case of fracture of synostosis and in case of stress fracture; regeneration medicine is also allowed. The surgical excision of the synostosis has a high index of reformation as described by Hanypsiak that of 15 patients surgically treated they have reported a regrowth in 27% of them^9^. We did not treat the synostosis, because the patient had shown no symptoms before fracture, the CT scan showed no fracture at the synostosis and during the reduction of the fracture the presence of synostosis had not created any problem. After one year, the patient did not report any symptoms that could be related to the presence of synostosis.

We think that the presence of synostosis has played a crucial role in the patient leg fracture considering the biomechanics without a significant trauma.

## IV. CONCLUSION

The presence of diaphyseal tibial fibular synostosis is uncommon and to our knowledge, this is the first case reported which shows the fractures of the tibia and fibula associated with an ipsilateral synostosis of the interosseous membrane. We think that the interosseous membrane plays an important role in biomechanics of the leg and if it is present can modify the biomechanics structure of the leg. However further studies are needed to define the role of the synostosis if it is present, and how to treat this structure in case it is damaged or not.

## Figures and Tables

**Tab. 1:** Personalized rehabilitation protocol

First step (II – IV week after trauma)	Second step (V – VIII week after trauma)	Third step (IX–XII week after trauma)
- Passive mobilization for ROM recovery of ankle - Recovery of muscle tone with isometric exercises - Active exercises with the use of mild teraband - Active mobilization of unloaded inflatable footrest	- Early ambulation training with the aid of crutches (after 1 month) - Proprioception exercises on inflatable and rigid board at partial load and early partial load on the (25 kg without discomfort) after IV weeks; after V weeks 35 kg load without discomfort, after VI weeks load without discomfort to 60kg and full static load 77 kg after 50 days from trauma	- complex proprioceptive exercises at full load - improvement of gait phases
